# The use of dupilumab in bronchiolitis obliterans after hematopoietic stem cell transplant

**DOI:** 10.1016/j.rmcr.2026.102508

**Published:** 2026-09-28

**Authors:** May Kyaw, Kevin Lu, Douglas Conrad, Tiffany Tanaka, Jeffrey Barry

**Affiliations:** a Department of Medicine, University of California San Diego, La Jolla, CA, United States; b Cleveland Clinic, Respiratory Institute, Cleveland, OH, United States; c Division of Blood & Marrow Transplantation, Department of Medicine, University of California San Diego, La Jolla, CA, United States; d Division of Pulmonary, Critical Care, Sleep Medicine, and Physiology, Department of Medicine, University of California San Diego, La Jolla, CA, United States

## Abstract

**Background:**

Bronchiolitis obliterans is a serious pulmonary manifestation of chronic graft-versus-host disease (cGVHD) following hematopoietic stem cell transplant (HSCT). Bronchiolitis obliterans is typically treated with a combination of inhaled steroids, azithromycin, montelukast and immunosuppressants. There are no curative treatments other than lung transplant, however, and additional therapies remain an unmet need.

**Case Presentation:**

Here we report two patients who developed bronchiolitis obliterans following HSCT. Both developed strong type 2 inflammatory markers including high eosinophil counts ≥ 1000 cells/mm^3^ and had history that was concerning for a possible concomitant asthma. Patient 1 had worsening symptoms after exposure to old wood during a construction project, and patient 2 had a history of asthma. Both patients were started on the anti-IL4 receptor monoclonal antibody, dupilumab, to target this type 2 inflammation. Both patients reported some symptomatic improvement but only had limited changes in their spirometry. Their courses were further complicated by multiple respiratory infections in the setting of immunosuppression for cGVHD.

**Conclusion:**

These cases highlight the unmet need for treatment in the bronchiolitis obliterans space and the potential role of type 2 inflammation in their pathogenesis. Given the complex nature of these cases and the infectious risks of immunosuppression, larger studies would be needed to better assess the potential role of dupilumab in this population.

## Highlights

• BOS represents a concerning pulmonary manifestation of cGVHD after HSCT.
• BOS cases require multidisciplinary care and often significant immunosuppression.
• Type 2 inflammation may play a significant role in cGVHD, including BOS.
• We present two cases where dupilumab was used in BOS with type 2 inflammation.
• Further studies on the use of dupilumab in BOS are needed.

## 1 Introduction

Hematopoietic stem cell transplant (HSCT) is a cornerstone therapy for many malignant hematologic disorders. Despite improvements in immunosuppression regimens, graft-versus-host disease (GVHD) remains a severe post-transplant complication associated with high morbidity and mortality occurring in 30-70% of transplant recipients [1]. Bronchiolitis obliterans syndrome (BOS) is a manifestation of chronic GVHD (cGVHD) and is estimated to develop in 6% of individuals with cGVHD [2]. The exact cause of BOS is unknown but is hypothesized to be from allorecognition of lung antigens resulting in intraluminal fibrosis of small terminal airways due to unregulated inflammation and epithelial disruption [3].

The clinical management of BOS requires multidisciplinary care. Newly diagnosed HSCT-associated BOS is treated with a combination of fluticasone propionate, azithromycin, montelukast (FAM) and a brief steroid burst with rapid taper [4]. Other agents targeting lymphocytes and cytokines have been used in the treatment of BOS, but therapy options for BOS remain an unmet need; there are no curative treatments other than lung transplantation with more recent studies showing a 5-year survival of only 40 to 50% [5].

Dupilumab, a monoclonal antibody targeting IL-4 and IL-13 through the IL-4 receptor (IL-4Rα), is used to treat moderate to severe asthma with an eosinophilic phenotype [6]. Both interleukins have been implicated in the pathogenesis of BOS [3]. Here, we report two patient cases with HSCT-associated bronchiolitis obliterans syndrome and possible concurrent bronchospasm treated with dupilumab.

## 2 Case series

### 2.1 Patient 1

A 45-year-old male with acute myeloid leukemia (AML) and myelodysplasia related changes underwent haploidentical allogeneic stem cell transplant. He received cyclophosphamide, tacrolimus and mycophenolate mofetil for GVHD prophylaxis. His tacrolimus taper was paused at 6 months post-transplant (PT) given concern for cGVHD with new development of eosinophilia (absolute eosinophil count 1800 cells/mm^3^ from 400 cells/mm^3^ prior) and musculoskeletal pain which promptly resolved with increased tacrolimus dose and a slower taper. Tacrolimus was tapered off by 9 months PT. Tacrolimus was restarted approximately two months later due to oral, ocular and possible liver cGVHD. During this time (11 months PT), he also developed dyspnea and cough with productive sputum. He had no known pulmonary history but was an active smoker (20 pack-year history) until his AML diagnosis. He was hospitalized for worsening respiratory status and found to be positive for metapneumovirus by PCR on bronchoalveolar lavage. His hospital course was complicated by acute respiratory distress syndrome requiring intubation. The patient's baseline pulmonary function tests (PFTs) prior to onset of his respiratory symptoms were unremarkable for obstruction with a normal ratio of forced expiratory volume in one second (FEV1) to forced vital capacity (FVC) of 85% and an FEV1 of 95% of the predicted value. His PFT one-month post-hospitalization demonstrated new obstructive lung disease with concern for BOS/cGVHD of the lung with a drop in FEV1 to 55% predicted with a FEV1/FVC ratio of 63%. (Fig. 1A). His CT scan showed new mosaic attenuation concerning for air trapping (Fig. 1B). He was started on high-dose prednisone at 100mg daily 14 months PT, as well as inhaled fluticasone, azithromycin and montelukast (FAM). His prednisone was tapered by 20mg approximately every 2 weeks until the patient reached 60mg daily, then by 10mg every 2 weeks until reaching 30mg daily, then by 5mg every 2 weeks until the patient reached 10mg. He was then maintained at 10mg daily from 19 months to 29 months PT. He had an initial modest increase in forced expiratory volume in 1 second (FEV1) that again worsened after subsequent influenza (25 months PT) and RSV (27 months PT) infections. At 26 months PT, he was noted to have expiratory wheezing in his bilateral lung bases and tiotropium-olodaterol was added to his fluticasone and prednisone taper, which was at 10mg daily at that time. He was started on ruxolitinib 27 months post-transplant for worsening BOS. Approximately three months after starting ruxolitinib, dupilumab was initiated at 200mg every 14 days given persistent wheezing superimposed on his bronchiolitis obliterans/lung cGVHD after his symptoms worsened following exposure to old wood during a construction project. It was felt asthma might be contributing to his decrease in FEV1, which was at 1.56 L (FEV1 41% predicted). His eosinophil count was less than 1500 cells/mm^3^ for 17 months prior to initiation of dupilumab. He was evaluated two months after starting dupilumab and reported improved symptoms with better activity tolerance and diminished cough. His FEV1 improved after 2 months to 1.92 L (51% predicted). His prednisone was ultimately discontinued 7 months after dupilumab initiation. He was later increased to the 300mg dose of dupilumab (43 months PT) due to feeling that the effects did not last the full two weeks.

**Fig. 1:**
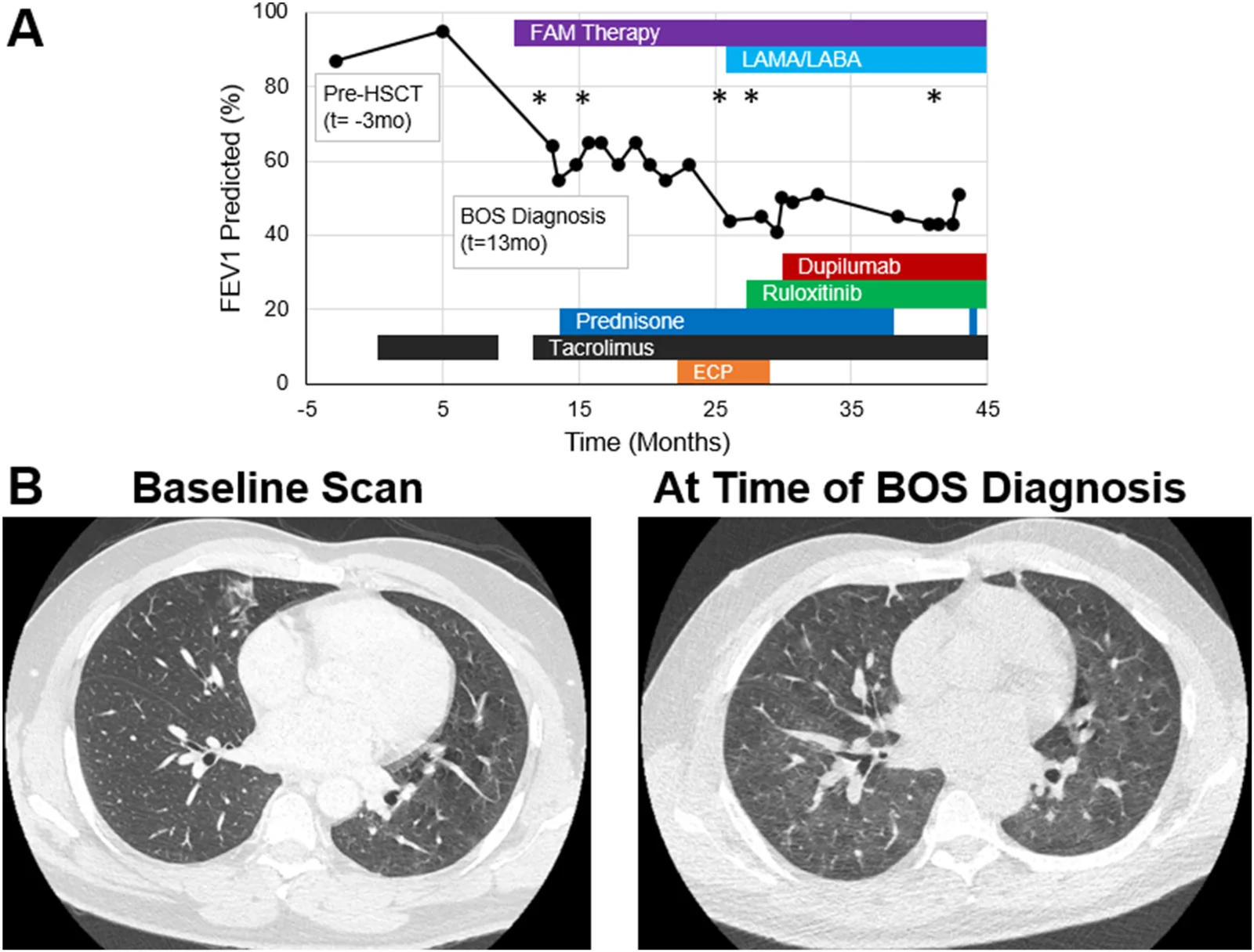
A) Patient 1: serial pulmonary function tests represented by percent predicted of FEV1 with notable treatment changes and infections. Time point at zero defined as day zero of the bone marrow transplant date. Asterisk (*) at timepoint represents episode of acute infectious respiratory illness. Colored bar length represents duration of treatment. B) CT imaging comparison of initial baseline one year prior to BOS presentation and at time of diagnosis. Radiologist read notable for mosaic attenuation with suggestion of air trapping. BOS = bronchiolitis obliterans syndrome. ECP = extracorporeal photopheresis. FAM = fluticasone, azithromycin, and montelukast. FEV1 = forced expiratory volume in 1 second. HSCT = hematopoietic stem cell transplant. LABA = Long-acting beta agonist. LAMA = Long-acting muscarinic antagonist.

#### 2.1.1 Patient 2

A 50-year-old male with HIV on antiretroviral therapy, asthma, a remote 26-pack-year smoking history and relapsed AML underwent matched unrelated donor allogeneic stem cell transplant with myeloablative conditioning with fludarabine and busulfan. At six months post-transplant, he was admitted for acute GVHD of the skin and liver. He was started on methylprednisolone as high as 80mg twice per day, ruxolitinib, and oral budesonide. He was also found to have vancomycin-resistant enterococcus pneumonia by bronchoalveolar lavage (BAL). Of note, his BAL had 5% eosinophils on cell count differential. His PFT at that time showed an FEV1 of 1.99L (54% predicted), down from the pre-transplant baseline of 3.29L (FEV1 89% predicted, with an FEV1/FVC of 63%) concerning for new BOS (Fig. 2A). Additionally, a CT of his chest demonstrated small multi-lobar nodular opacities and bronchial wall thickening (Fig. 2B). Azithromycin and montelukast were added to his inhaled steroid (ICS)/long-acting beta agonist (LABA) inhaler and he was continued on the methylprednisolone that was previously started for hepatic and cutaneous cGVHD. He was discharged after 1 month on a prolonged steroid taper with 55mg daily of prednisone, ruxolitinib, azithromycin, montelukast, and a budesonide-formoterol inhaler. He was started on abatacept from 8 to 13 months PT, and he completed the steroid taper at 9.5 months PT. The steroids were then resumed 17 months PT at 50mg of prednisone daily with a slow taper due to recurrence of his cGVHD of skin, mouth, and lungs. Ruxolitinib was also reinitiated at 18 months PT. Given his history of asthma and absolute eosinophilia (1100 cells/mm^3^), he was started on dupilumab 200mg every 14 days, approximately one year after his initial presentation for BOS (21 months PT). The patient had significant functional improvement. His FEV1 on initial PFT after starting dupilumab also improved slightly from 2.27 L (FEV1 65% predicted) to 2.37 L (68% predicted) though he had a subsequent decrease while hospitalized for a respiratory viral illness with superimposed pneumonia. His eosinophilia also normalized. Subsequently, the patient's course was complicated by GVHD flares requiring re-initiation of steroid tapers and multiple respiratory viral illnesses with subsequent drops in FEV1. He was trialed on belumosodil (29-30 months PT), axatilimab (34-39 months PT), ibrutinib (39-41 months PT), and abatacept (42-43 months PT) for his refractory cGVHD symptoms. His dupilumab dose was also increased to the 300mg dose at 40 months PT. He was changed to benralizumab shortly thereafter (42 months PT) due to a change in his insurance formulary. He had recurrent volume overload suspected to be either from his underlying diastolic heart failure and/or fluid retention related to prolonged steroid courses. Ultimately, he suffered a cardiac arrest following an elective left knee replacement and was transitioned by family to comfort care, two years after his initial presentation for BOS.

**Fig. 2:**
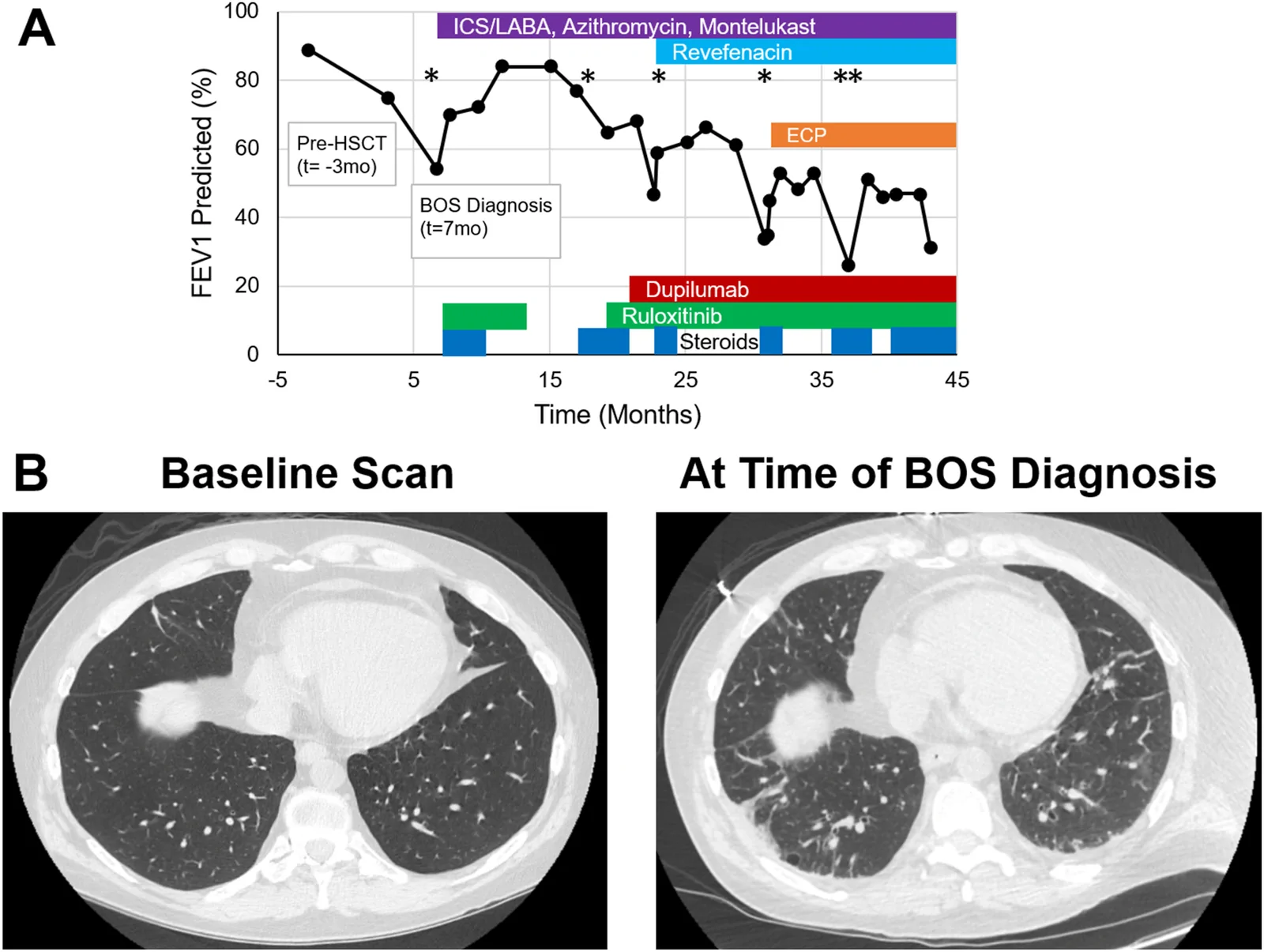
A) Patient 2: serial pulmonary function tests represented by percent predicted of FEV1 with notable treatment changes and infections. Time point at zerodefined as day zero of the bone marrow transplant date. Asterisk (*) at timepoint represents episode of acute infectious respiratory illness. Colored bar represents length represents duration of treatment with the dark blue bars representing systemic steroid courses. B) CT imaging comparison of initial baseline four months prior to BOS presentation and at time of diagnosis. Radiologist read notable for new bronchial wall thickening and small nodular opacities. BOS = bronchiolitis obliterans syndrome. ECP = extracorporeal photopheresis. FEV1 = forced expiratory volume in 1 second. HSCT = hematopoietic stem cell transplant. ICS = Inhaled corticosteroid. LABA = Long-acting beta agonist. LAMA = Long-acting muscarinic antagonist.

## 3 Discussion

In our case series, we report the use of dupilumab as an adjunctive therapy in patients with bronchiolitis obliterans and possible concomitant asthma with high type-2 (T2) inflammation after HSCT. We saw modest symptomatic improvement and increases in FEV1 after dupilumab initiation though this effect was confounded by subsequent respiratory infections. As previously described, dupilumab was tolerated well without significant side effects, but we did not see lung function increases on par with those seen in a previously reported case series of three BOS patients treated with dupilumab [7].

Both patients were initially treated for bronchiolitis obliterans with combination inhaled steroids, azithromycin, and montelukast. Previous studies have demonstrated that FAM therapy with a steroid course can stabilize lung function for the first three months, but additional therapies are needed to address progressive BOS despite FAM therapy [4]. It is notable that in both cases, this treatment had limited efficacy suggesting more severe disease. In addition, both patients were also treated with ruxolitinib, a Janus kinases (JAKs) 1 and 2 inhibitor, without meaningful improvement in lung function, demonstrating the severity of their progressive BOS. A recent phase 2 trial evaluating ruxolitinib in BOS showed that 92.3% of patients with BOS for more than 6 months were able to achieve stability of their lung function, defined as an absence of a ≥ 10% decrease in FEV1 [8]. DeFelipp et al. noted that clinical responses to ruxolitinib were more common in patients with mild or moderate disease and the median time to response was >7 months after starting. Patient 1 appears to have some slowing of his lung function decline after starting dupilumab and ruxolitinib, but patient 2 continued to progress. Both patients also had significantly higher spirometry prior to their BOS diagnoses and their obstruction did not fully reverse with treatment making it less likely to reflect solely bronchospasm or asthma.

Though the exact mechanism of possible benefit for dupilumab in bronchiolitis obliterans is unknown, there are several potential mechanisms that could be implicated, mainly driven by type-2 immune mediated responses. Multiple proinflammatory cytokines, including IL-13 and IL-4, are increased in the serum and T-cells of patients with cGVHD, and IL-13 and IL-4's effects would be mitigated by dupilumab [9]. Dupilumab also has been shown to reduce the type-2 T helper (Th2) cell proportion and reduce the programmed death-1 expression in regulatory T cells (Treg) in patients treated with dupilumab for chronic rhinosinusitis with nasal polyposis [10]. Both cell types may affect cGVHD with a relative decrease in Treg cells and T-cell polarization into Th2 cells both being associated with cGVHD. Previous reports have also demonstrated benefit in using dupilumab for atopic dermatitis (AD)-like GVHD and sclerotic-type cutaneous GVHD [11,12]. Elevated levels of IgE, Th2 cells and eosinophils may contribute to the T2 inflammation seen in atopic dermatitis (AD)-like GVHD [13]. Additionally, dupilumab was also well tolerated in both patients without adverse treatment-related effects such as hypereosinophilia. There was no evidence of new or worsening skin changes in our patient with cutaneous cGVHD upon initiation of dupilumab.

A limitation of this report is the number of cases due to the rarity of bronchiolitis obliterans. Additionally, recurrent respiratory infections and concomitant immunosuppressive therapies significantly confounded the direct effect of dupilumab on lung function trends. In both cases dupilumab may be treating a component of T2 inflammation associated with cGVHD as opposed to true concomitant asthma or eosinophilic chronic obstructive pulmonary disease (COPD). If the former, similar to the recent studies with dupilumab in COPD, this may be further evidence for the role of T2 inflammation overlaid across a spectrum of airway diseases, in addition to asthma, and supports use of dupilumab as a generally well tolerated therapeutic option to mitigate inflammation and bronchospasm [14,15]. Both patients had some smoking history with patient two having known asthma pre-transplant with a positive bronchodilator challenge pre-transplant. Though the patients did not have confirmatory pulmonary function testing showing bronchoreactivity after transplant, they did have significant variation in lung function which can be seen in asthma or with bronchospasm.

In these cases, we report the use of dupilumab in bronchiolitis obliterans after HSCT. Our patients had symptomatic respiratory improvement but not clear reversal of their obstructive lung disease. Dupilumab may be an appealing treatment option to address progressive BOS despite FAM therapy and ruxolitinib due to its steroid-sparing and non-immunosuppressant properties, but further investigation is warranted before adding it to the treatment armamentarium of bronchiolitis obliterans.

## CRediT authorship contribution statement

**May Kyaw:** Writing – review & editing, Writing – original draft. **Kevin Lu:** Writing – review & editing, Writing – original draft. **Douglas Conrad:** Writing – review & editing, Supervision, Conceptualization. **Tiffany Tanaka:** Writing – review & editing, Supervision. **Jeffrey Barry:** Writing – review & editing, Supervision, Conceptualization.

## Declaration of competing interest

The authors declare the following financial interests/personal relationships which may be considered as potential competing interests: Jeffrey Barry reports a relationship with Sanofi that includes: consulting or advisory. Jeffrey Barry reports a relationship with Regeneron Pharmaceuticals Inc that includes: consulting or advisory. Jeffrey Barry reports a relationship with Merck & Co Inc that includes: equity or stocks. Jeffrey Barry reports a relationship with AstraZeneca Pharmaceuticals LP that includes: consulting or advisory. Tiffany Tanaka reports a relationship with Bristol-Myers Squibb Company that includes: consulting or advisory. Tiffany Tanaka reports a relationship with Gilead Sciences Inc that includes: consulting or advisory. Tiffany Tanaka reports a relationship with Geron Corporation that includes: consulting or advisory. If there are other authors, they declare that they have no known competing financial interests or personal relationships that could have appeared to influence the work reported in this paper.
